# Anti‐Arrhythmic Effects of Linalool *via* Cx43 Expression in a Rat Model of Myocardial Infarction

**DOI:** 10.3389/fphar.2020.00926

**Published:** 2020-06-25

**Authors:** Jianlin Ke, Canzhan Zhu, Yuanyuan Zhang, Wenlong Zhang

**Affiliations:** ^1^ Second Affiliated Hospital of Shandong University of Traditional Chinese Medicine, Jinan, China; ^2^ Second Affiliated Hospital of Xi’an Jiaotong University, Xi’an, China; ^3^ Xi’an Honghui Hospital, Xi’an, China; ^4^ Shandong Provincial Hospital Affiliated to Shandong First Medical University, Jinan, China

**Keywords:** linalool, lavender, traditional medicine, arrhythmia, Cx43

## Abstract

**Background:**

Lavender is a traditional therapy for different heart symptoms including palpitation, which comprises an important symptom of cardiac arrhythmias. This experiment was designed to evaluate the antiarrhythmic effects of linalool using an experimental model of arrhythmia following myocardial infarction in rats. The underlying electrophysiological mechanism through cardiac connexin 43 (Cx43) expression was also investigated.

**Methods:**

Fifty male Sprague-Dawley rats were divided into five equal groups. The first group was considered as the normal control group; MI was induced by ligation of the left anterior descending artery (LAD) in the second group. The other three groups received metoprolol (100 mg/kg/day) or linalool (50 or 100 mg/kg/day) for seven days before LAD ligation. The arrhythmia score, isolated myocyte resting potential, histological changes, and cardiac Cx43 expression levels were evaluated.

**Results:**

In the MI group, there was a significant increase in the arrhythmia score but a marked decrease in resting membrane potential relative to the control; these changes were prevented by the administration of metoprolol or linalool. The histological changes were also minimized in the groups treated with these substances compared to the untreated MI group. The western blot and real-time PCR results showed that the protein expression of Cx43 in the infarct zone of the rat hearts was significantly higher in the MI groups receiving metoprolol or linalool compared with the untreated MI group.

**Conclusion:**

Linalool was shown to be able to dose-dependently decrease the incidence of arrhythmias in a rat model of myocardial infarction. We propose that the key mechanism behind this antiarrhythmic effect is probably the prevention of decreased Cx43 expression following MI.

## Introduction

Linalool is a naturally occurring monoterpene that comprises a key constituent of various aromatic plant essential oils (Kolouri et al., 2016), including that of the *Lavandula angustifolia* Mill., which is commonly known as the lavender (Caputo et al., 2018). This plant is traditionally named “Ostokhodus” in Persian medicine (Nasiri Lari et al., 2020), where it has been utilized as a natural remedy for various heart symptoms, including palpitation (Ershadifar et al., 2014). The lavender is presented as a medicinal plant for cardiac conditions in *Avicenna’s Treatise on Cardiac Drugs* (Faridi and Zarshenas, 2010; Mosavat et al., 2015).

Numerous studies have evaluated the effects of both lavender essential oil and its key constituent, linalool, on the cardiovascular system (Anjos et al., 2013). Linalool offers an antihypertensive effect according to the experimental models of hypertension (Anjos et al., 2013; Camargo et al., 2016). This substance is reported to induce vasorelaxation through the activation of guanylyl cyclase and K+ channels. Clinical studies also support the antihypertensive effects of lavender aromatherapy (Hwang, 2006; Gultom et al., 2016). Both linalool and lavender essential oil/extract have exerted cardioprotective effects in experimental models of myocardial ischemia and reperfusion injury (Wang et al., 2014; Ziaee et al., 2015; Zheng et al., 2017). The activation of Akt and the inhibition of GSK3b through the up-regulation of VEGF-B mRNA are suggested as potential pathways for linalool’s cardioprotective effects (Zheng et al., 2017).

Gap junctions between myocytes fulfill a significant role in cardiac electric conduction and synchronicity (Kang et al., 2008). These gap junctions are composed of two connexins rooted in the membranes of the connecting myocytes. Connexin 43 (Cx43) comprises the major gap junction protein in the heart ventricles (Dodge et al., 1998; Boengler and Schulz, 2017). A decrease in Cx43 expression leads to arrhythmias secondary to disordered cardiac electric conductivity. Experimental studies have elucidated an association between the cardiac Cx43 level and ischemic arrhythmias (Oyamada et al., 2001).

Despite the fact that several studies have evaluated the cardiovascular effects of linalool, the traditional claim about the substance’s effect on palpitations is yet to be scrutinized. The purpose of our study was to examine the antiarrhythmic effects of linalool using an experimental model of arrhythmias following myocardial infarction (MI) in rats. Furthermore, the underlying electrophysiological mechanism related to cardiac Cx43 expression was also investigated.

## Methods

### Animals

Fifty male Sprague-Dawley rats weighing 200–250 g were housed under a temperature of 23 ± 2°C and 60 ± 5% humidity with a twelve-hour light/dark cycle. Food and water were available ad libitum. Animal housing and the experimental procedures were compatible with the Guidelines for Ethical Conduct in the Care and Use of Animals. All procedures and experimental protocols were approved by the Animal Ethics Committee of Shandong University.

The rats were divided into five equal groups of ten. The first group was considered as the normal control group; MI was induced by ligation of the left anterior descending artery (LAD) in the second group. The other three groups received metoprolol (100 mg/kg/day) or linalool (50 or 100 mg/kg/day) for seven days ahead of LAD ligation. Linalool was purchased from Kanto Kagaku Co. Ltd (Tokyo, Japan),

### Myocardial Infarction (MI) Induction

As mentioned, MI was induced *via* LAD ligation (Sedighi et al., 2018). In brief, after achieving general anesthesia using sodium thiopental (60 mg/kg body weight, intraperitoneal), the animals underwent tracheal intubation and were ventilated with a tidal volume of 1.5 cc/kg at a rate of 60–70 breaths/min. Then, an incision was made in the left fourth intercostal space of the chest. The pericardium was slowly torn, and a 0.6 silk thread was carefully passed around the LAD and fastened. After 30 min of ischemia, the LAD suture was removed and reperfusion of the ischemic myocardium was allowed for 120 min. The control group underwent an identical procedure but without the actual tying of the suture.

### ECG Recording and Arrhythmia Scoring

In the rats, the electrocardiogram of limb lead II was recorded, with the arrhythmias being assessed as described in a previous study (Wang et al., 2011). The scores presented in **Table 1** were used to quantify the incidence and duration of arrhythmias. In each group, the mean frequency and duration of ventricular arrhythmias and the mean RR interval were analyzed by the Kubios HRV electrocardiogram analysis software.

**Table 1:** Scoring system used to grade the arrhythmias of the rats.

Score	Definition
0	No arrhythmia
1	<10 s PVC and/or VT
2	11–30 s PVC and/or VT
3	31–90 s PVC and/or VT
4	91–180 s PVC and/or VT, <10 s reversible VF
5	> 180 s PVC and/or VT, >10 s reversible VF
6	Irreversible VF

VF, Ventricular fibrillation; VT, ventricular tachycardia; PVC, premature ventricular contraction.

### Isolation of Myocytes

The myocytes were separated from the rat hearts following a method described in a related study (Yang et al., 2008). In brief, the rats were sacrificed after seven days of treatment, MI induction, and ECG recording. The rat hearts were removed for retrograde perfusion *via* the coronary circulation. The Krebs-Ringer solution (pH 7.4, 95% O2) was used for perfusion with a flow rate of 13 ml/min under heart temperature (37°C). The peri-ischemic cardiac tissue was minced in the storage solution, before the isolated myocytes were centrifuged for electrophysiological recording. The whole-cell patch-clamp method was employed for the electrophysiological recording (Kornreich, 2007).

### Assessment of the Pathologic Changes

Three rats from each group were used for the pathologic study. A transjugular injection of 2,3,5-triphenyltetrazolium chloride was made to allow better visualization of the infarct area. Full-thickness sections from the myocardium were obtained for pathologic studies. Formalin (10%) was used for fixation with routine laboratory sample processing.

### Cardiac Cx43 Western Blotting and Real-Time PCR

At the end of the perfusion experiments, the ventricular tissue of the hearts was isolated and frozen immediately. On the day of preparing the ventricular sarcolemma, the stored ventricles were homogenized with an ultrasound homogenizer in a hypotonic membrane buffer containing 1 mM of 1,10-phenanthroline, 1 mM of iodoacetamide, 1 mM of pepstatin A, and 0.4 mM of phenylmethylsulfonyl fluoride (PMSF) (Sonics & Material Inc., Jencons Ltd., Germany). The western blot technique was performed according to the method described in another study (Yuan et al., 2011). The prepared samples were incubated with the primary antibodies (Cx43 polyclonal antibody; 1:1000 dilution, LSBio, USA) and glyceraldehude-3-phosphatedehydrogenase (GAPDH; 1:2000, LSBio) at 4°C for 12 h. Horseradish peroxidase-conjugated immunoglobulin IgG (1:1500, LSBio) was applied as the second antibody for 1 h at room temperature. The ECL kit (LSBio) was used for visualization of the blots, with signal analysis being performed using the ChemiDoc Imaging System (Bio-Rad, USA).

The real-time PCR assay of Cx43 was performed as described in a related study (Zhang et al., 2014). The RNA was extracted from the cardiac tissue using the reagents and transcribed to complementary DNA. The real-time PCR reaction solution consisted of forward and reverse primers for the final reaction as well as complementary DNA. Glyceraldehyde-3-phosphate dehydrogenase (GAPDH) was used as a negative control to test for genomic DNA. Specific primers for Cx43 and GAPDH were designed using primer BLAST. The nucleotide sequences of the primer pair selected for GAPDH were 5′-TGGCAAAGTGGACATCGTTG-3′ (forward) and 5′ TGGCGTGGACAGTGGTCATAAGTC-3′ (reverse), with an expected amplified product of 467 base pairs. The nucleotide sequences of the primer pair selected for Cx43 were 5′-TCGTGTCGTTGGTGTCTCTTG-3′ (forward) and 5′-GAGGAGCAGCCATTGAAATAAGC-3′ (reverse).

### Statistical Analysis

The data were analyzed by SPSS 25.0 (IBM Inc., USA). The results were expressed as mean values ± standard deviation. The one-way analysis of variance (ANOVA) test was used for the comparison of outcomes among groups. The Bonferroni test was employed for adjusting the multiple comparisons test to prevent data from incorrectly appearing to be statistically significant. *P* values of less than 0.05 were considered as statistically significant. The relevant plots were created with Graph Pad Prism 8 (Graph Pad Software Inc., USA).

## Results

### Arrhythmia Score

**Figure 1:**
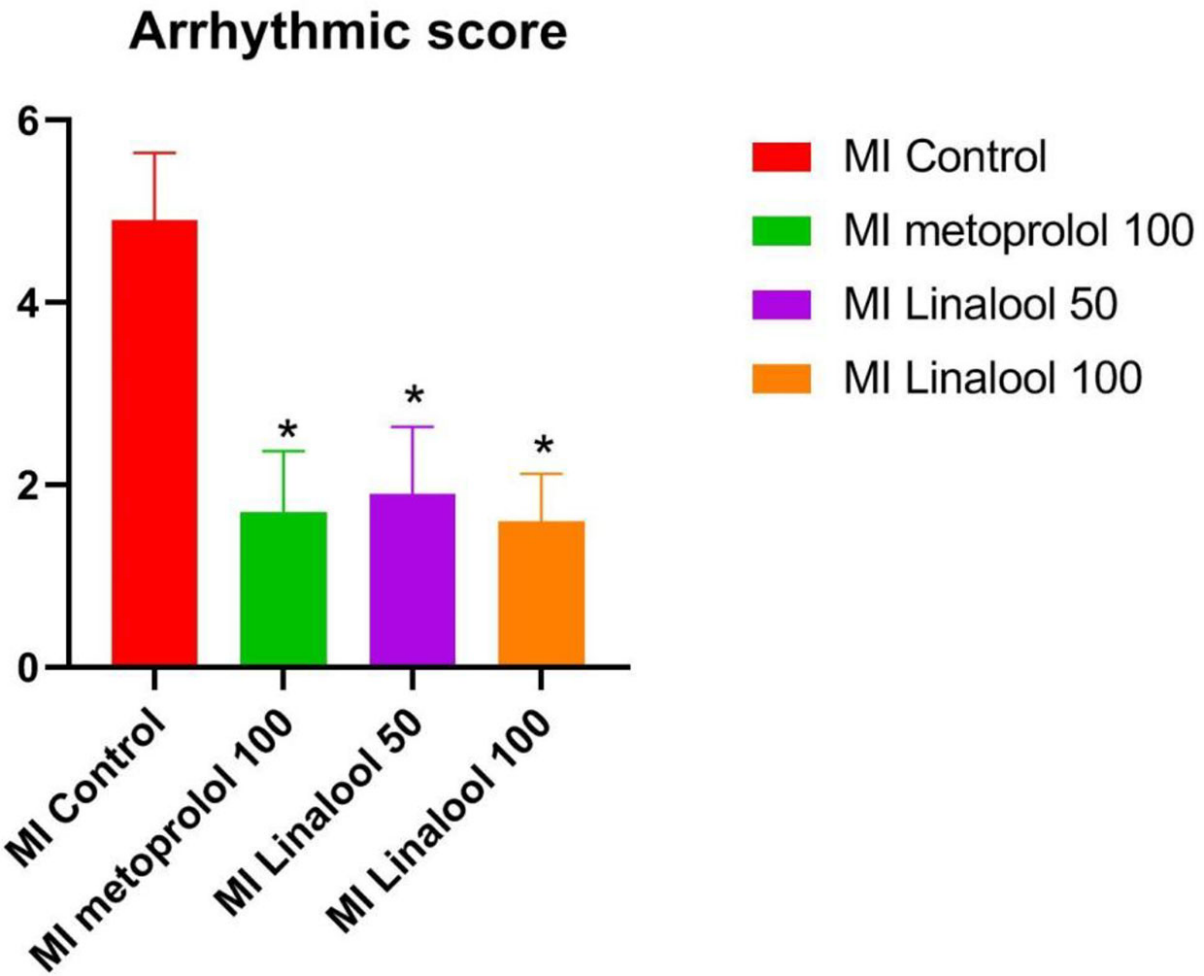
Effects of linalool on arrhythmic scores for the cardiac arrhythmias recorded in the different study groups. *p < 0.05 vs. MI group.

**Figure 2:**
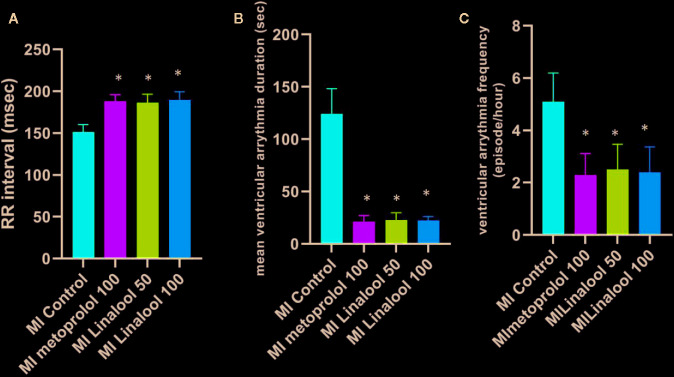
Effects of linalool on RR intervals, mean frequency and duration of ventricular arrhythmia in the study groups. *p < 0.05 vs. MI group.

No arrhythmias were recorded in the control group, leading to a score of zero. Different types of arrhythmias were recorded in the MI group, which achieved a score of 4.90 ± 0.74 (p < 0.01 compared with control). As depicted in **Figure 1**, the arrhythmia scores were significantly lower in the MI groups treated with 100 mg/kg/day of metoprolol (1.70 ± 0.67; p < 0.01), 50 mg/kg/day of linalool (1.90 ± 0.74; p < 0.01) or 100 mg/kg/day of linalool (1.60 ± 0.52; p < 0.01) relative to the untreated MI group. As demonstrated in **Figure 2**, there was a significantly longer RR interval and significantly lower mean frequency and duration of ventricular arrhythmias in the groups treated with linalool or metoprolol compared with the untreated MI group (p < 0.01). There was no significant difference among the treated groups in the Bonferroni corrected multiple comparison test (p = 1.00).

### Resting Membrane Potential

**Figure 3:**
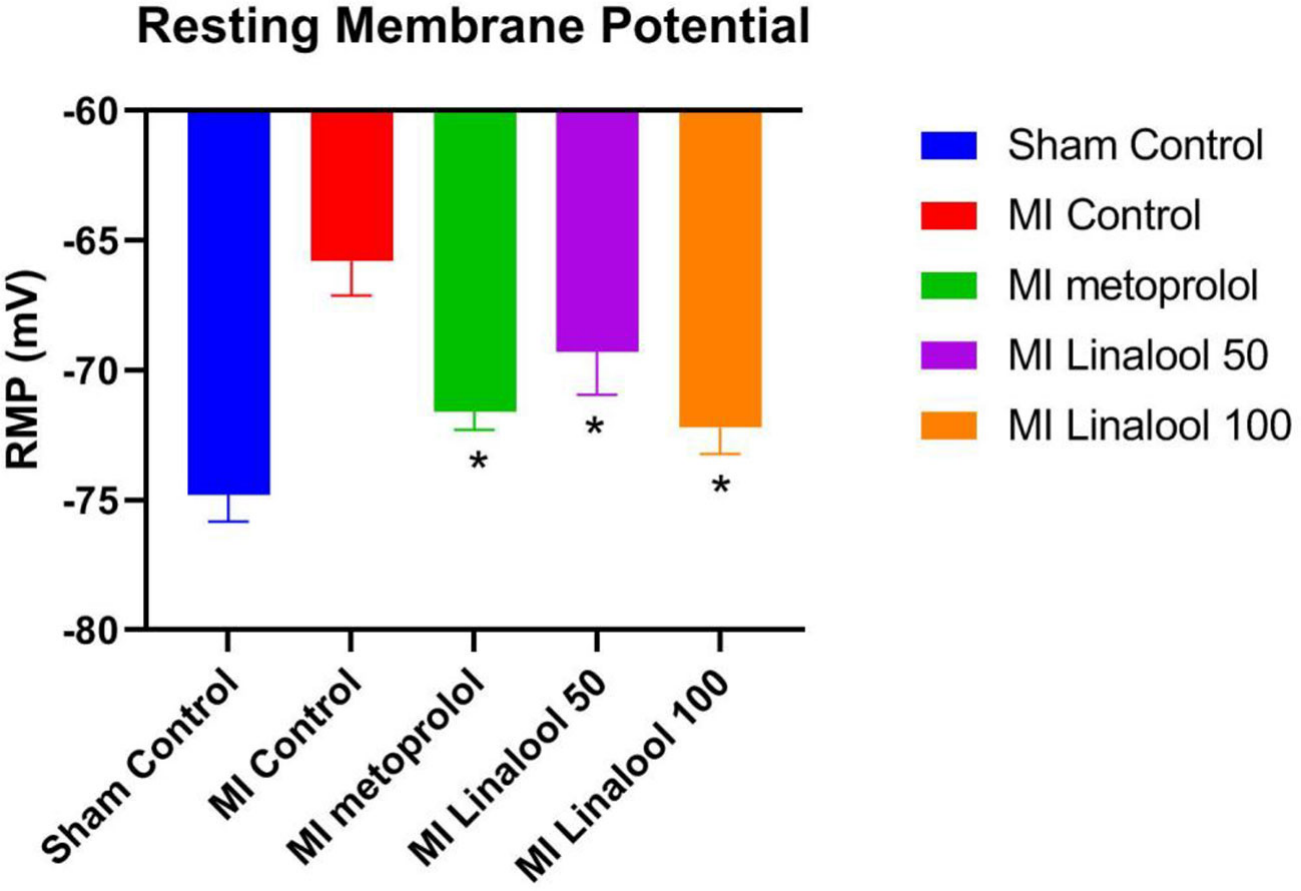
Effects of linalool on the rest membrane potential (RMP) in isolated rat ventricular myocytes in the different study groups. *p < 0.05 vs. MI group.

The cardiac myocytes’ resting membrane potential was significantly depolarized from −74.80 ± 1.03 mV in the control group to −65.80 ± 1.32 mV in the MI group (p < 0.01). This depolarization was markedly prevented by 100 mg/kg/day of metoprolol (−71.60 ± 0.70 mV; p < 0.01), 50 mg/kg/day of linalool (−69.30 ± 1.64; p < 0.01), or 100 mg/kg/day of linalool (−72.20 ± 1.03; p < 0.01) when compared against the untreated MI group (**Figure 3**).

### Histology

**Figure 4:**
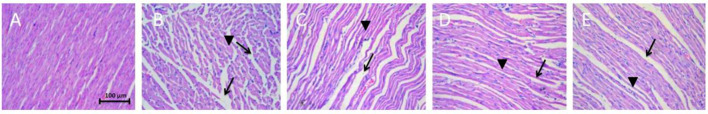
Effects of linalool on histological changes resulting from anterior coronary artery ligation in the different study groups. **(A)** Sham control group with no ischemic change. **(B)** Myocardial infarction group without any treatment with extensive myofilament disarray and loss (→) and swelling of myocardial cells (►). **(C–E)** Metoprolol, Linalool 50 and 100 mg/kg treated groups, respectively. Decreased myofilament disarray, loss and swelling is observed.

In the rats with MI, the anterior region of the heart showed extensive cell edema and myofilament disarray and loss. However, this pathologic change was significantly less in the hearts of the rats in the metoprolol and linalool treatment groups (**Figure 4**).

### Western Blot

**Figure 5:**
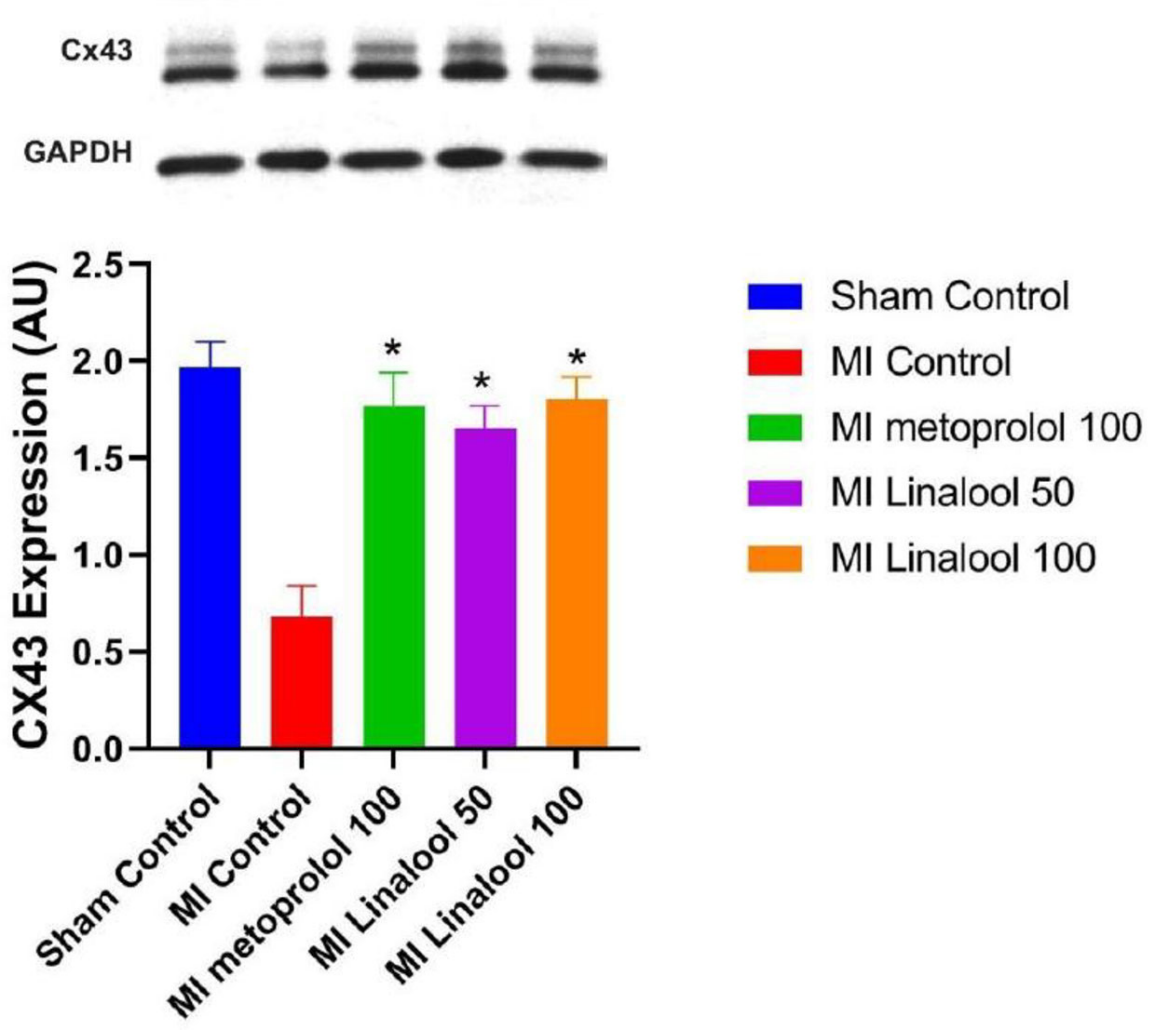
Effects of linalool on western blot of Cx43 protein expression levels in the different study groups. *p < 0.05 vs. MI group.

The level of Cx43 protein expression was evaluated *via* the western blot technique, which revealed significantly less Cx43 expression in the infarcted zone of the MI group (0.68 ± 0.16; p < 0.01) compared against the normal control (1.97 ± 0.13). This decrease was considerably prevented using 100 mg/kg/day of metoprolol (1.77 ± 0.17; p < 0.01), 50 mg/kg/day of linalool (1.65 ± 0.12; p < 0.01), or 100 mg/kg/day linalool (1.80 ± 0.12; p < 0.01) relative to the untreated MI group (**Figure 5**).

### Real-Time PCR Analysis

**Figure 6:**
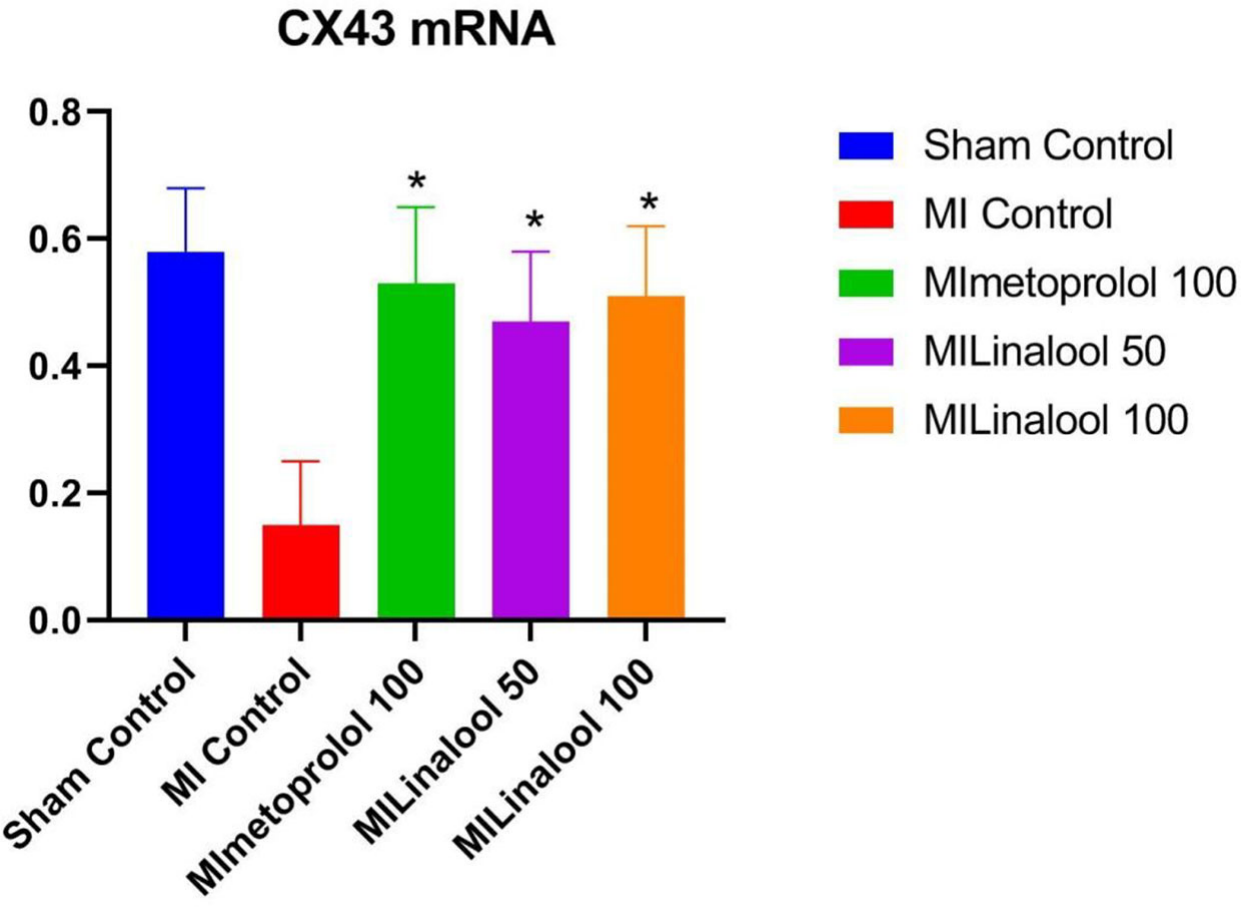
Effects of linalool on Cx43 mRNA expression levels in the different groups. *p < 0.05 vs. MI group.

The expression of Cx43 mRNA was evaluated by real-time PCR analysis. According to the results, there was a significantly lower rate of Cx43 mRNA expression in the infarct zone of the MI group (0.15 ± 0.10; p < 0.01) compared with the control group (0.58 ± 0.10). This decrease was significantly deterred by 100 mg/kg/day of metoprolol (0.53 ± 0.12; p < 0.01), 50 mg/kg/day of linalool (0.47 ± 0.11; p < 0.01), or 100 mg/kg/day of linalool (0.51 ± 0.11; p < 0.01) when compared against the untreated MI group (**Figure 6**).

## Discussion

This study revealed that like metoprolol, linalool can exert antiarrhythmic effects in a dose-dependent manner in a rat model of MI. This effect was related to the prevention of a decrease in Cx43 expression in the infarcted zone.

It is previously demonstrated that experimental myocardial ischemia in rats induces different types of rhythm disturbances including ventricular fibrillation (VF), ventricular tachycardia (VT), and premature ventricular contraction (PVC) (Tribulova et al., 2008). These arrhythmias are attributed to delayed after depolarizations in surviving Purkinje fibers, which cause an increase in the automaticity of these fibers in the ischemic area (Scheinman, 2009; Di Diego and Antzelevitch, 2011).

Metoprolol is a beta-receptor blocker that belongs to class II of the antiarrhythmic drugs. Beta-blockers prevent sudden cardiac death caused by malignant ventricular arrhythmias in MI (Yan et al., 2007; Haugaa et al., 2010). Abundant evidence exists regarding the preventive effect of metoprolol on Cx43 degradation as an important mechanism for the antiarrhythmic effect of this drug in MI. This pathway can recover gap junction communication, leading to improved conduction velocity. These changes ultimately reduce susceptibility to ventricular arrhythmias (Zhou et al., 2017; Zhai et al., 2018).

Linalool, also known as linalyl alcohol, linaloyl oxide, β-linalool, and allo-ocimenol (Kazemi et al., 2016), is a natural product with a wide range of pharmacologic effects on the cardiovascular system (Santos et al., 2011). This substance is found in different medicinal plants including *Cinnamomum tamala* (Chanotiya and Yadav, 2010)*, Cannabis sativa* (Fischedick et al., 2010)*, Cannabis indica* (Naz et al., 2017)*, Ocimum basilicum* (Duman et al., 2010)*, Artemisia vulgaris* (Judžentienė and Buzelytė, 2006), and *Humulus lupulus* (Miyashita and Sadzuka, 2013). However, *L. angustifolia* Mill. (lavender) is the most popular plant that contains linalool as its main ingredient (Ciobanu et al., 2012; Caputo et al., 2018). This plant is traditionally used as a natural remedy for palpitation, the most important symptom of arrhythmias (Ershadifar et al., 2014). It has previously been shown that lavender essential oil can modulate T-type calcium channels in HEK-293T cells, which comprises an important pathway in the prevention of neuronal excitability (El Alaoui et al., 2017). However, the mechanism behind the antiarrhythmic function of linalool had not previously been investigated.

The current results introduce the possible antiarrhythmic mechanisms of linalool. Connexin 43 is an important structural protein in cardiac gap junctions; it fulfills a significant role in cell coupling during electric signal conduction (Mostafavi et al., 2015). Changes in Cx43 expression are associated with different types of arrhythmias following myocardial infarction (Fana et al., 2014). Our experiment showed that linalool can prevent decreases in Cx43 messenger RNA and protein levels following myocardial infarction. Hence, our findings suggest that the anti-arrhythmic mechanism of linalool is likely to be similar to that of metoprolol in the prevention of Cx43 degradation.

The mechanism by which linalool prevents Cx43 degradation has not been investigated in detail. The prevention of ischemic injury is a potential means through which linalool can affect the Cx43 level. It is recognized that myocardial ischemia can result in Cx43 degradation (Hatanaka et al., 2004). Hypoxia of cardiomyocytes can decrease Cx43 expression and cause downregulation and internalization of Cx43 at gap junctions, ultimately decreasing the total cellular Cx43 content (Schulz et al., 2015). Linalool is known to be able to prevent myocardial ischemic injuries (Zheng et al., 2017). In the experimental model of myocardial ischemia, linalool inhibited the inflammatory response, prevented oxidative stress, and increased the level of vascular endothelial growth factor B, thereby providing protection against ischemia-induced cell death and apoptosis (Zheng et al., 2017).

Our study also showed that the significant depolarization that occurred in the MI group (compared with the control) was prevented by linalool administration. Depolarization can increase myocyte excitability by shifting the resting membrane potential to a less negative value (Parham et al., 2006). Hence, besides the prevention of Cx43 degradation, maintaining the resting membrane potential may be another mechanism that explains the observed antiarrhythmic effect of linalool. This effect on myocardial resting membrane potential may also explain the previously shown preventive effect of linalool on myocardial ischemic injuries (Zheng et al., 2017). Ischemic injury can damage the myocyte membrane channels, which leads to depolarization in the myocyte resting membrane potential, resulting in increased excitability and arrhythmias (Shaw and Rudy, 1997).

In summary, linalool was shown to be able to dose-dependently decrease the incidence of arrhythmias in a rat model of myocardial infarction. We propose that the key mechanism behind this antiarrhythmic effect is probably the prevention of decreased Cx43 expression following MI. Our research shows that linalool may be a promising candidate for the pharmacotherapy of ventricular arrhythmias related to myocardial infarction.

## Data Availability Statement

The raw data supporting the conclusions of this article will be made available by the authors, without undue reservation.

## Ethics Statement

The animal study was reviewed and approved by The Ethical Board of Shandong University.

## Author Contributions

WZ designed and supervised the work and wrote the first draft of the manuscript. CZ performed the electrophysiological studies. YZ conducted the western blot and real-time PCR. All authors contributed to the article and approved the submitted version.

## Conflict of Interest

The authors declare that the research was conducted in the absence of any commercial or financial relationships that could be construed as a potential conflict of interest.
